# Outcomes of Percutaneous Endoscopic Gastrostomy in Huntington's Disease at a Tertiary Center

**DOI:** 10.1002/mdc3.14130

**Published:** 2024-06-09

**Authors:** Mena Farag, Annabelle Coleman, Harry Knights, Michael J. Murphy, Sangeerthana Rajagopal, Alexiane Touzé, Maryam Shoai, Cara Hearst, Desiree M. Salanio, Edward J. Wild, Sarah J. Tabrizi

**Affiliations:** ^1^ Huntington's Disease Centre, UCL Queen Square Institute of Neurology University College London London United Kingdom; ^2^ The National Hospital for Neurology and Neurosurgery (NHNN), Queen Square London United Kingdom; ^3^ Department of Neurodegenerative Diseases, UCL Queen Square Institute of Neurology University College London London United Kingdom

**Keywords:** clinically assisted nutrition and hydration, dysphagia, Huntington's disease, percutaneous endoscopic gastrostomy, weight loss

## Abstract

**Background:**

Clinically assisted nutrition and hydration via percutaneous endoscopic gastrostomy (PEG) is a therapeutic option to ameliorate the difficulties associated with enhanced catabolism, weight loss, and dysphagia in Huntington's disease (HD).

**Objectives:**

The objective is to provide insights into demographics, staging (Shoulson‐Fahn), complications, weight trajectories, and survival rates in people with HD (pwHD) who underwent PEG.

**Methods:**

This retrospective study included 705 consecutive pwHD who attended our HD clinic between July 2006 and March 2024, of whom 52 underwent PEG. A control group (n = 52), comprising pwHD without PEG, were closely matched for sex, stage, age, CAG length, and disease burden score at PEG. The study was registered as a service evaluation at the National Hospital for Neurology and Neurosurgery.

**Results:**

PEG prevalence was 15.0% (n = 52/347) among manifest pwHD: 4.8% (n = 3/62) for Stage 3; 33.3% (n = 16/48) for stage 4; and 44.1% (n = 30/68) for stage 5. Commonest indications were dysphagia, weight loss, and inadequate oral intake. Complications included chest infection, tube dislodgement, and peristomal and skin infections. Modeling of weight trajectories after PEG found no difference between PEG and non‐PEG groups. Mortality rate was 34.6% (n = 18/52) in the PEG and 36.5% (n = 19/52) in the non‐PEG groups (*P* = 0.84). Treatment duration (until study endpoint or death) was 3.48 years (interquartile range = 1.71–6.02; range = 0.23–18.8), with 65.4% (n = 34/52) alive at the study endpoint.

**Conclusion:**

PEG in pwHD at‐risk for weight loss may help slow weight loss. Prospective studies are required to strengthen PEG decision‐making in pwHD. PEG survival was much longer than other dementias, highlighting the need to consider PEG independently in pwHD.

Huntington's disease (HD) is an autosomal dominant neurodegenerative condition caused by an expanded CAG trinucleotide repeat in the *HTT* gene, resulting in the expression of mutant huntingtin protein (mHTT). The pathogenesis of HD is largely attributed to the detrimental toxic gain‐of‐function properties of mHTT, leading to neuronal dysfunction and death.^1^ Clinically, HD is characterized by progressive behavioral disturbance, cognitive decline, and movement disorder, notably chorea.

The progression of the disease is insidious, typically spanning 15 to 20 years from the time of diagnosis to death, with the onset of symptoms usually within the prime of adult life.^2^ In ~5% of cases, symptoms manifest before the age of 21 and are classified as juvenile‐onset Huntington's disease (JoHD).^2^ Despite the progress of research into disease‐modifying treatments, HD continues to be a relentlessly progressive disorder that remains incurable. To date, there are no approved treatments to delay onset or slow progression of this devastating condition.^3^ A multidisciplinary approach forms the mainstay of management, which incorporates supportive and symptomatic treatments aimed to alleviate behavioral, mood, and motor symptoms.

Weight loss is common in HD and is multifactorial. Excessive movements result in an enhanced catabolic state meaning that normal, or even high, calorie intakes are insufficient to sustain weight.^4^ Reduced appetite, related to the psychiatric state,^5^ may compound this, alongside the development of dysphagia later in the disease course.^6^ Dysphagia precipitates a higher risk of pulmonary aspiration, a leading cause of mortality in HD.^7^ Weight loss, driven by the symbiotic interplay between the catabolic nature of HD, psychiatric symptoms, and dysphagia, has implications for prognosis, with one study finding that higher baseline body mass index (BMI) is associated with slower disease progression, independent of CAG length.^8^ Therefore, it is crucial to monitor the nutritional status of people with HD (pwHD).

Clinically assisted nutrition and hydration can serve as a therapeutic option to ameliorate the difficulties associated with enhanced catabolism, weight loss, and dysphagia in HD.^4^ Percutaneous endoscopic gastrostomy (PEG) feeding is a minimally invasive intervention involving insertion of a small tube through the abdominal wall directly into the stomach, circumventing the compromised swallowing mechanism. Although PEG tube usage does not reduce the risk of aspiration,^9^, ^10^ the primary objective is to sustain weight (either by slowing weight loss, maintaining, or increasing weight by comparison to individuals without PEG), thereby maintaining physiological stability, which may otherwise compound disease progression. Beyond nutritional support, hydration and reliable medication administration are additional reasons to consider PEG insertion. Choices about PEG insertion in pwHD are individualized. Decisions can be integrated within Advance Care Planning (ACP) discussions, including the formalization of an advance statement and/or an advance decision to refuse treatment to ensure that management aligns with the individual's expressed preferences and wishes.^11^

There is scant literature on gastrostomy feeding in pwHD, with a lack of data regarding pre‐ and post‐PEG insertion outcomes, specifically relating to staging of disease, timing of the procedure, complications, survival, and the role of ACP in PEG decisions. This information is important for pwHD, families, and healthcare providers when considering PEG insertion. This retrospective chart review provides insights into outcomes related to PEG insertion in the largest cohort of HD patients studied to date, from a single tertiary center over 17 years.

## Methods

### Standard Protocol Approvals, Registrations, and Patient Consents

In line with guidelines of the Health Research Authority (https://www.hra-decisiontools.org.uk/research), the study was classed as part of service evaluation, formally registered (registration number: 40‐202,324‐SE) and approved by the Queen Square Quality and Safety Team, affiliated with University College London Hospitals National Health Service (NHS) Foundation Trust. Additionally, we adhered to the STROBE cohort reporting guidelines.^12^

### Data Acquisition

This retrospective study involved selection of case notes from a cohort of 788 consecutive patients who attended the multidisciplinary Huntington's disease clinic at the National Hospital for Neurology and Neurosurgery (NHNN), Queen Square. Patients within the neurogenetic clinic arm of the service who were considered at risk because of family history, but had not yet undergone genetic testing at their latest follow‐up (n = 68) were excluded. Given that JoHD represents a distinct cohort from adult‐onset HD, with different disease trajectories and clinical needs, people with JoHD (n = 15) were also excluded from the analysis. The analysis focused on eligible patients (n = 705) reviewed in the clinic between July 10, 2006 and March 7, 2024.

Data collected included date of birth, sex, CAG repeat length (derived from neurogenetics reports where available), details regarding the presence of PEG tube (including indication, date of insertion, and complications), type of residence at last follow‐up review, and whether the individual underwent ACP review in our dedicated ACP clinic. Information from each clinic visit regarding documented weight, complications related to PEG, and disease staging (Shoulson‐Fahn) were also collected. Mortality data, specifically date of death, was also collected. For those with CAG repeat length data available and age at PEG insertion, a disease burden score (DBS) at PEG insertion was calculated retrospectively using the Langbehn formula (DBS = age × [CAG – 35.5]).^13^

Disease staging was extracted from the clinical notes where documented by clinicians. Staging followed the Shoulson‐Fahn criteria,^14^ as categorized by the Unified Huntington's Disease Rating Scale (UHDRS) Total Functional Capacity (TFC).^15^ Stages ranged from stage 1 (early stage, with full to near‐full functional capacity) to stage 5 (advanced stage, with maximum care requirements and dependence). Staging is standardized using the UHDRS TFC scale as follows: TFC 11 to 13 corresponds to stage 1, TFC 7 to 10 to stage 2, TFC 4 to 6 to Stage 3, TFC 1 to 3 to stage 4, and TFC 0 to stage 5.^15^ Numeric stages directly derived from the clinical notes were used for the analysis. Note the Huntington's Disease Integrated Staging System (HD‐ISS),^16^ which incorporates an evidence‐based research framework for staging HD that has not been integrated into routine clinical practice, is not described.

To ensure validity of the conclusions derived from the patients who underwent PEG insertion (n = 52), the control group (n = 52) comprised of individuals with HD without PEG closely matched with the PEG cohort for sex, disease stage, age, CAG repeat length, and DBS at the time of PEG insertion (in this order).

ACP is the process of undertaking anticipatory discussions about preferences for future care in the United Kingdom. This is documented in the medical record as a formal discussion and aims to ensure future care respects patient wishes, especially in situations where an individual is at‐risk of losing mental capacity to make specific decisions in the future. Our HD service runs a specialized ACP clinic^11^ and attendance at this clinic was required to categorize an individual as having engaged in a formal ACP discussion.

### Data Availability Policy and Statement

Anonymized data available on reasonable request from any qualified investigator.

### Statistical Analysis

Statistical analyses were performed with Stata/MP 18.0. Before analysis, the normality of each variable was assessed to ensure the appropriate selection of statistical tests for inter‐group comparisons. Group demographic differences in age and DBS between the PEG and non‐PEG groups were conducted using an independent two‐sample *t* test assuming equal variances. Group differences between CAG length were assessed using a Mann‐Whitney *U* test because of non‐normally distributed data. Age differences between disease stage groups were assessed using the Mann‐Whitney *U* test because of disease subgroup age being non‐normally distributed. Sex, ACP, and mortality *P*‐values were derived from χ^2^ testing. Fisher's exact testing was used to detect differences in disease stage and type of residence between PEG and non‐PEG groups.

For the weight analysis, a mixed‐effects regression model was performed to assess the impact of PEG on weight trajectory. Fixed effects were time with PEG, group (with PEG vs. without PEG), sex, age at PEG insertion, disease stage at PEG insertion, and baseline weight (weight at PEG insertion); and random effects were the individual and time with PEG. Note that weights at the point of PEG insertion, and after, were included in this model. Where baseline weight (at PEG insertion) was not available, closest weight up to 2 years before PEG insertion was used. Where no weight was available up to 2 years before PEG insertion, the closest weight up to 2 years after PEG insertion was used. pwHD with missing data in any fixed or random effects were not incorporated in the model.

A separate mixed effects regression model was performed to assess for possible differences in weight trajectory between the PEG and non‐PEG groups before the decision to insert/not insert PEG. Fixed effects were time before PEG decision, group (for PEG or not for PEG), sex, age at PEG insertion, disease stage at PEG insertion, and baseline weight (here, first weight recorded). Note that weights at the point of PEG insertion were not included in this model. pwHD with missing data in any fixed or random effects were not incorporated in the model.

For the mixed effects regression models, the Bonferroni method was used to correct for multiple comparisons. Six coefficients were used, and therefore, the corrected significance threshold was 0.05/6 = 0.0083.

## Results

### Study Population and Clinical Characteristics

Of 705 consecutive patients reviewed in the multidisciplinary HD clinic between July 10, 2006 and March 7, 2024, 52 patients (7.4%) underwent PEG insertion. Of the total cohort of pwHD without PEG (n = 653), the control group (n = 52), comprising individuals with HD without PEG, were closely matched with the PEG cohort for sex, disease stage, age, CAG repeat length, and DBS at the time of PEG insertion. There were no statistically significant differences between the two groups with respect to any of the aforementioned variables (Table 1). This indicates a high degree of similarity between the two groups, being well‐matched and suitable for comparative analysis.

**TABLE 1:** Study population and clinical characteristics

Characteristics	PEG (n = 52)	Non‐PEG (n = 52)	*P* value
Sex, n (%)			
Female	25 (48%)	25 (48%)	1.0000
Male	27 (52%)	27 (52%)	
Age at PEG insertion (y)			
Median	51.5	55.5	0.6047
Mean ± SD	53.1 (10.96)	54.2 (11)	
IQR	54–61	47–62	
CAG repeat length			
n	24	34	0.5407
Median	45	44	
Mean ± SD	46 (5.4)	45.2 (3.9)	
IQR	42.5–47.5	43–47	
Disease burden score			
At PEG insertion	24	34	0.4632
Median	479	480	
Mean ± SD	501 (124)	480 (90)	
IQR	420–547	423–536	
Disease stage at PEG insertion, n (%)			
Stage 5	28 (54%)	28 (54%)	1.0000
Stage 4	14 (27%)	14 (27%)	
Stage 3	7 (13%)	7 (13%)	
Unknown	3 (6%)	3 (6%)	
Disease stage at last follow‐up review, n (%)			
Stage 5	40 (77%)	30 (57%)	0.1592
Stage 4	7 (13%)	16 (31%)	
Stage 3	2 (4%)	3 (6%)	
Unknown	3 (6%)	3 (6%)	
Disease stage age at PEG insertion, y; median (IQR)			
Stage 5	55 (47–61)	56 (53–62)	0.3802
Stage 4	51 (42–62)	53 (44–66)	0.8180
Stage 3	49 (38–53)	52 (43–53)	0.8981
Unknown	50 (49–68)	47 (41–65)	0.2752
Indication(s) for PEG insertion			
Dysphagia	36 (34.3%)	–	–
Weight loss	31 (29.5%)		
Inadequate oral intake	20 (19.0%)		
Hospitalization for pneumonia	6 (5.7%)		
Psychological factors^a^	5 (4.8%)		
Administration of medication	3 (3.9%)		
Unknown	4 (3.8%)		
Mortality (n dead)			
n (%)	18 (34.6%)	19 (36.5%)	0.8377
Duration from date of PEG insertion to death (days)			
Median	954	–	–
Mean (SD)	1131 (1048)		
IQR	346–1286		
ACP review, n (%)			
Yes	10 (19.2%)	11 (21.2%)	0.8070
No	42 (80.8%)	41 (78.8%)	
Type of residence at last review			
Care home	25	30	<0.0005
24‐hour carer	10	0	
Package of care	11	10	
Family carer	6	2	
Own home	0	10	

Values are means (SD), n (%), or median (IQR). Overall age and DBS between the PEG and non‐PEG groups were assessed using an independent two‐sample *t* test of equal variances. CAG repeat length between PEG and non‐PEG was assessed using Mann‐Whitney *U* test. Sex, ACP, and mortality *P*‐values were derived from χ^2^ testing. Disease stage differences between PEG and non‐PEG groups at PEG insertion and at last follow‐up were assessed using Fisher's exact test. Type of residence at last review was assessed using Fisher's exact test. Age differences between disease stage groups were assessed using the Mann‐Whitney *U* test.

^a^ Indications were associated with psychological factors such as fear of eating (n = 4), where PEG tube insertion was considered beneficial to alleviate the psychological distress associated with eating. Additionally, there was one case where perseverative speech and preoccupation with HD were contributing to impaired eating habits. Note total number of indications exceeds number of PEG tube insertions in our cohort because multiple indications were often documented for a single case.

Abbreviations: PEG, percutaneous endoscopic gastrostomy; SD, standard deviation; IQR, interquartile range; ACP, advance care planning; DBS, disease burden score; HD, Huntington's disease.

Excluding cases where PEG was inserted, but disease staging was not documented in the medical records (n = 3), the prevalence of PEG insertion was observed as follows: 4.8% (n = 3/62) for Stage 3, 33.3% (n = 16/48) for stage 4, and 44.1% (n = 30/68) for stage 5 HD. Therefore, the cumulative prevalence of PEG insertion in our cohort of HD patients stages 3 to 5 was 27.5% (n = 48/178). Among stages 1 to 5, indicative of clinically manifest HD, the overall prevalence was 15.0% (n = 52/347).

The primary indications for PEG insertion, as documented in the medical records, included dysphagia (n = 36, 34.3% of total indications), weight loss (n = 31, 29.5%), and inadequate oral intake (n = 20, 19%). Notably, six patients underwent PEG tube insertion while they were hospitalized for pneumonia. In three cases, administration of medication was documented as an indication for PEG insertion. Psychological factors (n = 5, 4.8%) also contributed to decision‐making for PEG insertion. For example, four patients expressed fear of eating, where PEG insertion was considered beneficial to alleviate the psychological distress associated with eating. Additionally, one instance highlighted perseverative speech and preoccupation with HD contributing to impaired eating habits.

In the PEG group, 19.2% (n = 10) had engaged in formal ACP discussions documented in the ACP clinic. Similarly, in the non‐PEG group, 21.2% (n = 11) underwent ACP review in the clinic.

Significant differences emerged in the living arrangements and type of residence between the two groups at last follow‐up. Among the PEG group, the distribution included 25 in care homes, 10 receiving 24‐hour care, 11 with established packages of care, six under family care, and none residing in their own homes. Conversely, the distribution in the non‐PEG group was different: 30 in care homes, none receiving 24‐hour care, 10 supported by packages of care, two under family care, and 10 managing to live in their own homes.

### Complications Documented in pwHD Following PEG Insertion

Data on complications documented in the PEG group are shown in Table 2. The most common complications were chest infection (n = 18, 26.5%) and inadvertent tube dislodgement (n = 18, 25.6%), followed by peristomal infection (n = 11, 16.2%), and then skin irritation (n = 8, 11.8%). One patient experienced a significant early complication necessitating surgical intervention with washout, gastropexy and fixation of the gastrostomy. The remaining complications were non‐acute as documented during follow‐up, as derived from medical records.

**TABLE 2:** Complications documented in pwHD following PEG insertion

Complication	Frequency (n)	%
Chest infection	18	26.5
Inadvertent tube dislodgement	18	26.5
Peristomal infection	11	16.2
Skin irritation	8	11.8
Peristomal leakage	3	4.4
Reflux	2	2.9
Bleeding stoma site	1	1.5
Granulation tissue	1	1.5
Sleep disturbance	1	1.5
Peritonitis^a^	1	1.5
Pneumoperitoneum^a^	1	1.5
Surgical intervention^a^	1	1.5
Tube blockage	1	1.5
Tube fracture	1	1.5
Total	68	

Values are n (%).

^a^ Complications were associated with acute complications in one case following PEG tube insertion. The remaining complications were non‐acute as documented during follow‐up, as derived from medical records.

Abbreviations: pwHD, people with Huntington's disease; PEG, percutaneous endoscopic gastrostomy.

### Longitudinal Weight Outcomes in pwHD with and without PEG

A total of 89/104 (85.6%) pwHD had a weight measured at any time point. For the regression modeling before PEG, 69/104 (66.3%) had a weight recorded before PEG insertion/non‐insertion. A total of 65/104 (62.5%) were included in the regression modeling after PEG insertion after removal of pwHD with missing data‐points, and four were missing disease stage at PEG insertion. For the 65 pwHD used in the model, the mean number of longitudinal weight recordings per individualwas 3.8 (range = 1–14). There was no difference in weight trajectory between the PEG and non‐PEG groups, before the PEG decision (*P* = 0.48, coefficient = 0.97, CI = −1.71 to 3.65). However, time to PEG decision did have an effect, which survived correction for multiple comparisons (*P* = 0.003, coefficient = ‐0.46, CI = −0.77 to −0.16). Baseline weight (first weight recorded) also had an effect (*P* < 0.0005, coefficient = 0.73, CI = 0.61–0.86). Sex, age at PEG insertion, and disease stage at PEG insertion did not have an effect (*P* values, coefficients, and CI are in Table S1).

For the regression modeling before PEG, 71/104 (68.2%) had a weight measured at or after PEG insertion/non‐insertion. A total of 52/104 (50%) were included in the regression modeling after PEG insertion after removal of pwHD with missing data‐points. Two were missing disease stage at PEG insertion, 15 were missing baseline weight (at PEG insertion), and two were missing both. For the 52 pwHD used in the model, the mean number of longitudinal weight recordings per individual was 3 (range = 1 to 11). PEG insertion was not associated with a difference in weight trajectory (*P* = 0.51, coefficient = 1.12, confidence interval [CI] = –2.24 to 4.48). Time with PEG did havean effect (*P* = 0.046, coefficient = –0.82, CI = ‐1.62 to ‐0.01), but this did not survive Bonferroni correction for multiple comparisons (*P* threshold = 0.0083). Baseline weight (at PEG insertion) had an effect, which survived correction for multiple comparisons (*P* < 0.0005, coefficient = 0.79, CI = 0.66–0.92). Sex, age at PEG insertion, and disease stage at PEG insertion did not have an effect (P values, coefficients, and CI are in the Supporting information (Table S2)).

**FIG. 1:**
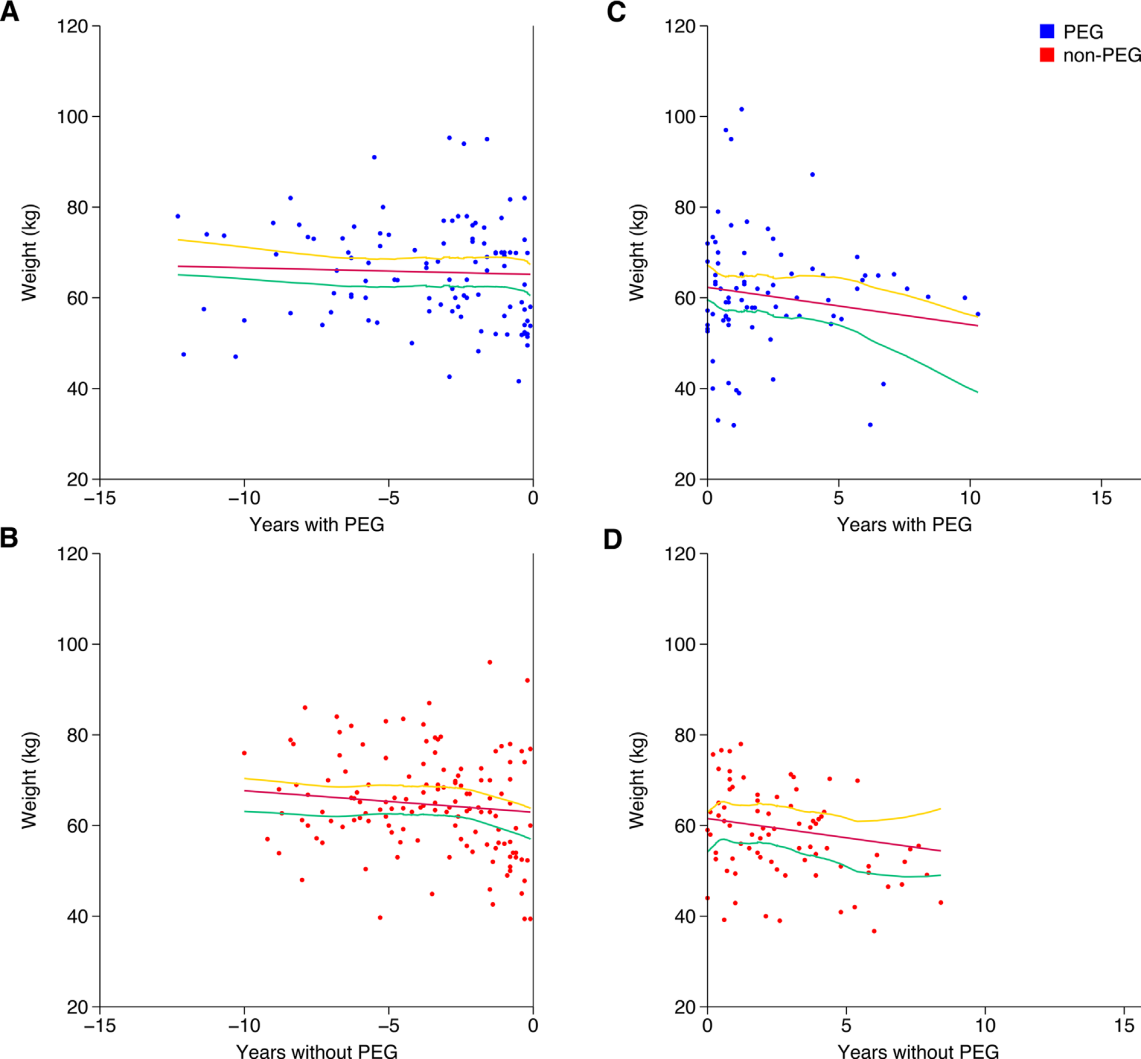
Comparative weight trajectories between percutaneous endoscopic gastrostomy (PEG) and non‐PEG groups over time. Weight against years with/without PEG. (**A,C**) represent the PEG group (blue dots); (**B**,**D**) represent the non‐PEG group (red dots). Years without PEG in controls are defined by the date of PEG insertion in their matched counterpart with PEG. Mixed‐effects regression models (red lines) with 95% confidence intervals (CI) (lower green; upper yellow) are also depicted. Two mixed‐effects regression models were created: one for pre‐PEG (**A,B**) and one for post‐PEG (**C,D**). PEG status did not impact weight trajectory in the pre‐PEG (*P* = 0.48, coefficient = 0.97, CI = −1.71 to 3.65) or post‐PEG (*P* = 0.51, coefficient = 1.12, CI = −2.24 to 4.48) models.

Weight trajectories are depicted in Figure 1. Model outputs are described in detail in the Tables S1 and S2.

### Mortality Outcomes in pwHD with and without PEG

Data on mortality outcomes for the PEG and non‐PEG groups are shown in Table 1. A total of 34.6% of patients in the PEG group (n = 18/52) died by the study endpoint, compared to 36.5% (n = 19/52) in the matched non‐PEG group. The difference in mortality outcomes between both groups did not reach statistical significance (*P* = 0.838). Median treatment duration (until study endpoint or death) was 3.48 years (interquartile range (IQR) = 1.71–6.02 years; range = 0.23–18.8 years), although 65.4% (n = 34/52) were alive at the study endpoint, meaning this is an underestimate of true treatment duration.

**FIG. 2:**
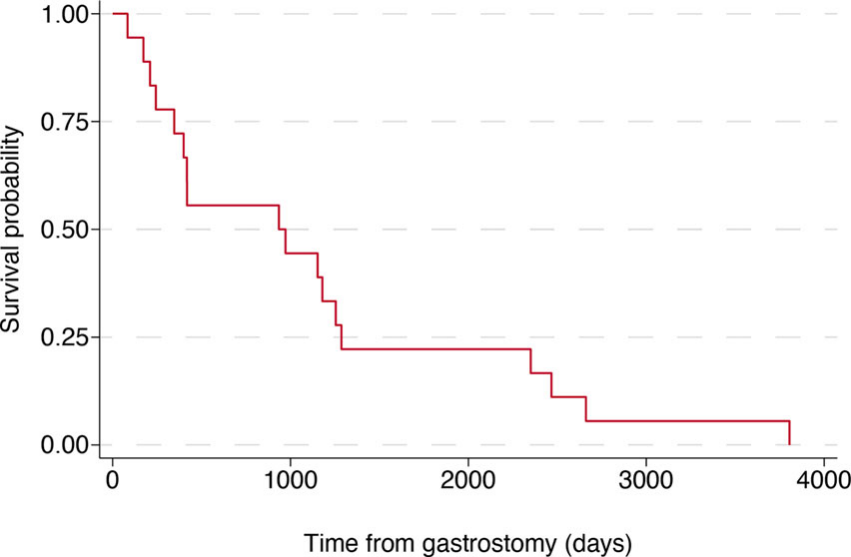
Kaplan–Meier survival curve for percutaneous endoscopic gastrostomy (PEG) group. This survival plot pertains to the subgroup analysis of people with Huntington's disease (pwHD) with PEG who died (n = 18/52). Median duration from date of PEG insertion to date of death was 2.61 years (interquartile range = 0.95–3.55 years) with a median age at death of 55.4 years. Note this survival duration is less than the whole cohort because it represents the subset of individuals who died before the study endpoint.

A subgroup analysis of the pwHD with PEG who died (18/52) was also performed, finding that those who died had a median age at death of 55.4 years and a median survival duration of 2.61 years (IQR = 0.95–3.55 years) from the date of PEG insertion to the date of death. Note this survival duration is less than the whole cohort because it represents the subset of individuals who died before the study endpoint. A Kaplan–Meier survival curve for the PEG group is shown in Figure 2.

## Discussion

This chart review represents an extensive retrospective longitudinal study of pwHD with PEG in a tertiary center, offering essential insights into demographics, disease staging (Shoulson‐Fahn), complications, objective weight outcomes, and survival rates. In our cohort, pwHD with PEG were predominantly in the advanced stages of the disease, which is expected because PEG insertion is often considered when excess movements and swallowing difficulties become more pronounced later in the disease course. The overall prevalence of PEG in individuals with clinically manifest HD across stages 1 to 5 in our cohort was 15% (n = 52/347). This is lower than the prevalence of 26.4% (n = 39) reported in an earlier retrospective study of 148 pwHD, although this study did not provide information on the clinical stages of study participants involved in the analysis.^17^

The indications for PEG insertion in this study reveal several key findings. The commonest indications were dysphagia, inadequate oral intake (relative to calorific requirement), and weight loss. The term “inadequate oral intake (relative to calorific requirement)” was phrased to represent individuals who were treated with PEG intervention because of concerns over inadequate oral intake, whether or not this had resulted in measurable weight loss to date. It is worth noting that the inter‐relation between these indications is complex and variable in HD. For example, inadequate oral intake may or may not be because of dysphagia and may or may not have already resulted in weight loss. These observations align with the outcomes of a previous retrospective analysis of case records involving 14 HD patients who underwent PEG insertion, where the leading indications were difficult oral feeding and significant weight loss.^18^

Managing the care and follow‐up of pwHD with PEG tubes presents a range of challenges. In our patient cohort, chest infection and inadvertent tube dislodgement emerged as the most common documented complications. Notably, choreiform movements were identified as a potential factor in tube dislodgement, as medical records indicated the challenges posed by chorea in the administration of medications and the feeding process. Similarly, a retrospective review of case records from 14 patients with HD who underwent PEG insertion also revealed that 35.7% (n = 5) experienced early complications related to tube dislodgement.^17^ The reported complications of tube blockage, tube fracture, peristomal infection, and skin irritation underscore the importance of diligent post‐PEG tube management.

The primary goal of PEG is to administer nutrition and hydration directly into the gastrointestinal tract and does not reduce the risk of aspiration,^8^, ^9^ which is one of the most frequent overall causes of death in pwHD.^19^ Our study revealed 26.5% (n = 18) events of chest infection documented after PEG insertion. These occurrences, however, do not differentiate between aspiration as a complication directly related to the PEG procedure and aspiration secondary to the progression of HD. Unfortunately, a comparative analysis of chest infection rates in the matched non‐PEG group was hindered because of limited documentation of such events in the medical records of the non‐PEG group.

Current literature on the nutritional and survival benefit of PEG in HD is limited with one retrospective study reporting that PEG tube insertion increases the length of life, but has no impact on nutritional measures or weight.^17^ In our cohort, we have shown that there was no difference in the trajectory of weight change between the PEG and matched non‐PEG groups. When interpreting this finding, it must be noted that the PEG group were either experiencing weight loss or were felt to be at‐risk for weight loss (with inadequate oral intake and/or dysphagia). The absence of a decrease in weight in the PEG group, therefore, suggests that PEG intervention in pwHD with inadequate oral intake, dysphagia, and/or weight loss may help prevent subsequent weight loss at a greater rate than those not requiring a PEG. However, it is important to note that the weight data is highly heterogeneous, which likely reflects the retrospective design and inability to accurately control for other influencing factors, including the concomitant use of neuroleptic medication for chorea, with effects on appetite, the precise feeding regimen, and inaccuracies in weight measurements taken in clinic. Further prospective studies are required to provide the certainty needed to impact clinical decision‐making.

The analysis of mortality rates between the closely matched PEG and non‐PEG groups revealed no statistically significant differences between the two groups. This outcome suggests that PEG intervention and the maintenance of weight is not associated with an increase in mortality rates due to complications arising from PEG insertion.

The survival analysis for the total number of patients who underwent PEG insertion in our HD cohort reveals additional important insights into their outcomes. Median treatment duration (until study endpoint or death) was 3.48 years (IQR = 1.71–6.02 years; range = 0.23–18.8 years). It must also be noted that 65.4% (n = 34) of the PEG group were alive at the study endpoint, and therefore, true treatment duration is likely even longer. In another study of mortality following PEG insertion in Parkinson's disease and related neurodegenerative conditions, including progressive supranuclear palsy, multisystem atrophy, dementia with Lewy bodies, and vascular parkinsonism, the median survival period across all groups was 422 days (1.16 years).^20^ These findings highlight the difference in PEG survival outcomes between pwHD and other neurodegenerative diseases. Conceptually, this difference may be because of the hyperkinesia and heightened catabolic state that characterizes early HD, and the decision to perform PEGs in this context without significant weight loss or dysphagia. This may mean PEGs are inserted earlier in the disease course, allowing for longer survival times. This finding highlights the need to consider HD independently from other dementias and neurodegenerative conditions when considering PEG.

The main limitation of this study is the retrospective design with incomplete data capture, particularly for weight. This limited the sample size of the mixed effect regression modeling because not all individuals had weights measured and baseline weights were not accurately imputable for all pwHD. Complications may not have been fully documented or recorded, likely resulting in under‐reported complications post‐PEG insertion in our cohort. Furthermore, the lack of patient‐reported outcome measures after PEG insertion impedes assessing post‐PEG quality of life.

This study expands the knowledge base on prevalence, indications, and outcomes of PEG insertion in a large single‐center cohort. Our findings reveal no statistically significant differences in terms of weight change or mortality in our PEG cohort, compared to an age, sex, and disease stage matched cohort. This study provides support to the notion that intervention with PEG tube in pwHD may aid in maintaining weight in those susceptible to weight loss without raising mortality rates. However, further prospective studies are required to provide the certainty needed to impact clinical decision‐making. Survival with PEG was much longer than other dementias and neurodegenerative conditions, highlighting the need to consider HD independently in ACP.

## Author Roles

(1) Research project: A. Conception, B. Organization, C. Execution; (2) Statistical Analysis: A. Design, B. Execution, C. Review and Critique; (3) Manuscript Preparation: A. Writing of the First Draft, B. Writing of the Second Draft, C. Review and Critique.

M.F.: 1A, 1B, 1C, 2A, 2B, 2C, 3A, 3B

A.C.: 1B, 1C, 2A, 2B, 2C, 3A, 3B

H.K.: 1B, 1C, 2A, 2B, 2C, 3B

M.J.M.: 1C, 2C, 3A

S.R.: 1C, 2C, 3A

A.T.: 1C, 2C, 3A

M.S.: 2C

C.H.: 1B, 3A

D.M.S.: 1B, 3A

E.J.W.: 2C, 3C

S.J.T.: 2C, 3C

All authors were involved in final approval of the revised manuscript.

## Disclosures

**Ethical Compliance Statement:** In line with guidelines of the Health Research Authority (https://www.hra-decisiontools.org.uk/research), the study was classed as part of service evaluation, formally registered (Registration Number: 40‐202,324‐SE) and approved by the Queen Square Quality and Safety Team, affiliated with University College London Hospitals NHS Foundation Trust. Informed patient consent was not necessary for this work. We confirm that we have read the Journal's position on issues involved in ethical publication and affirm that this work is consistent with those guidelines.

**Funding Sources and Conflicts of Interest:** M.F., M.J.M., and S.J.T. received research grant funding from the Wellcome Trust (223,082/Z/21/Z). S.J.T. also received funding from the UK Dementia Research Institute (DRI) that receives its funding from DRI, funded by the UK Medical Research Council (MRC), Alzheimer's Society, and Alzheimer's Research UK. E.J.W. reports grants from UK MRC, CHDI Foundation, European Huntington's Disease Network (EHDN), and F. Hoffmann‐La Roche. S.R. received funding from Guarantors of Brain. The salaries of A.C. and A.T. were supported by a MRC Career Development Award (MR/W026686/1). The authors declare that there are no conflicts of interest relevant to this work.

**Financial Disclosures for the previous 12 months:** In the previous 12 months S.J.T. has received research grant funding from the CHDI Foundation, the National Institute for Health and Care Research (NIHR), and the UK MRC. Through the offices of University College London (UCL) Consultants, a wholly owned subsidiary of UCL, S.J.T. has undertaken consultancy services in the past 12 months for Alnylam Pharmaceuticals, Annexon, Arrowhead Pharmaceuticals, Ascidian Therapeutics, Atalanta Therapeutics, Design Therapeutics, F. Hoffman‐La Roche, Iris Medicine, Latus Bio, LifeEdit, Novartis Pharma, Pfizer, Prilenia Neurotherapeutics, PTC Therapeutics, Rgenta Therapeutics, Takeda Pharmaceuticals, UniQure Biopharma, and Vertex Pharmaceuticals. In the past 12 months, University College London Hospitals NHS Foundation Trust, S.J.T.'s host clinical institution, received funding to run clinical trials for F. Hoffman‐La Roche, Novartis Pharma, PTC Therapeutics, and UniQure Biopharma. E.J.W. discloses consultancy and advisory services with services to entities including Alnylam Pharmaceuticals, Annexon, Cumulus Neuroscience, F. Hoffmann‐La Roche, Ionis Pharmaceuticals, PTC Therapeutics, Remix Therapeutics, Takeda Pharmaceuticals, Teitur Trophics, Triplet Therapeutics, UniQure Biopharma, and VICO Therapeutics.

## Supporting information


**Table S1.** Mixed effects regression model output for before PEG. Weight (kg); timetopeg = time (years) before PEG insertion; group = PEG vs non‐PEG; sex = male vs female; pegage = age (years) at PEG insertion/non‐insertion; stagepeg = stage (Shoulson and Fahn staging) at PEG insertion/non‐insertion; basepre = baseline weight (kg, first weight recorded).
**Table S2.** Mixed effects regression model output for after PEG insertion. Weight (kg); timetopeg = time (years) after PEG insertion; group = PEG vs non‐PEG; sex = male vs female; pegage = age (years) at PEG insertion/non‐insertion; stagepeg = stage (Shoulson and Fahn staging) at PEG insertion/non‐insertion; basepre = baseline weight (kg, weight at PEG insertion where available, otherwise imputed – see Methods section for details).

## Data Availability

Anonymised data available upon reasonable request from any qualified investigator.
