# Supplementary material for: Outcomes of Percutaneous Endoscopic Gastrostomy in Huntington's Disease at a Tertiary Center
Source: Mov Disord Clin Pract. 2024 Jun 9;11(8):998–1007. doi: 10.1002/mdc3.14130 (PMC11329559; doi:10.1002/mdc3.14130)
Supplement: Supplementary file 1 — Table S1. Mixed effects regression model output for before PEG. Weight (kg); timetopeg = time (years) before PEG insertion; group = PEG vs non‐PEG; sex = male vs female; pegage = age (years) at PEG insertion/non‐insertion; stagepeg = stage (Shoulson and Fahn staging) at PEG insertion/non‐insertion; basepre = baseline weight (kg, first weight recorded). Table S2. Mixed effects regression model output for after PEG insertion. Weight (kg); timetopeg = time (years) after PEG insertion; group = PEG vs non‐PEG; sex = male vs female; pegage = age (years) at PEG insertion/non‐insertion; stagepeg = stage (Shoulson and Fahn staging) at PEG insertion/non‐insertion; basepre = baseline weight (kg, weight at PEG insertion where available, otherwise imputed – see Methods section for details). [file MDC3-11-998-s001.docx]

**Supplementary:**

**Supplemental Table 1:**

**Mixed effects regression model output for before PEG**


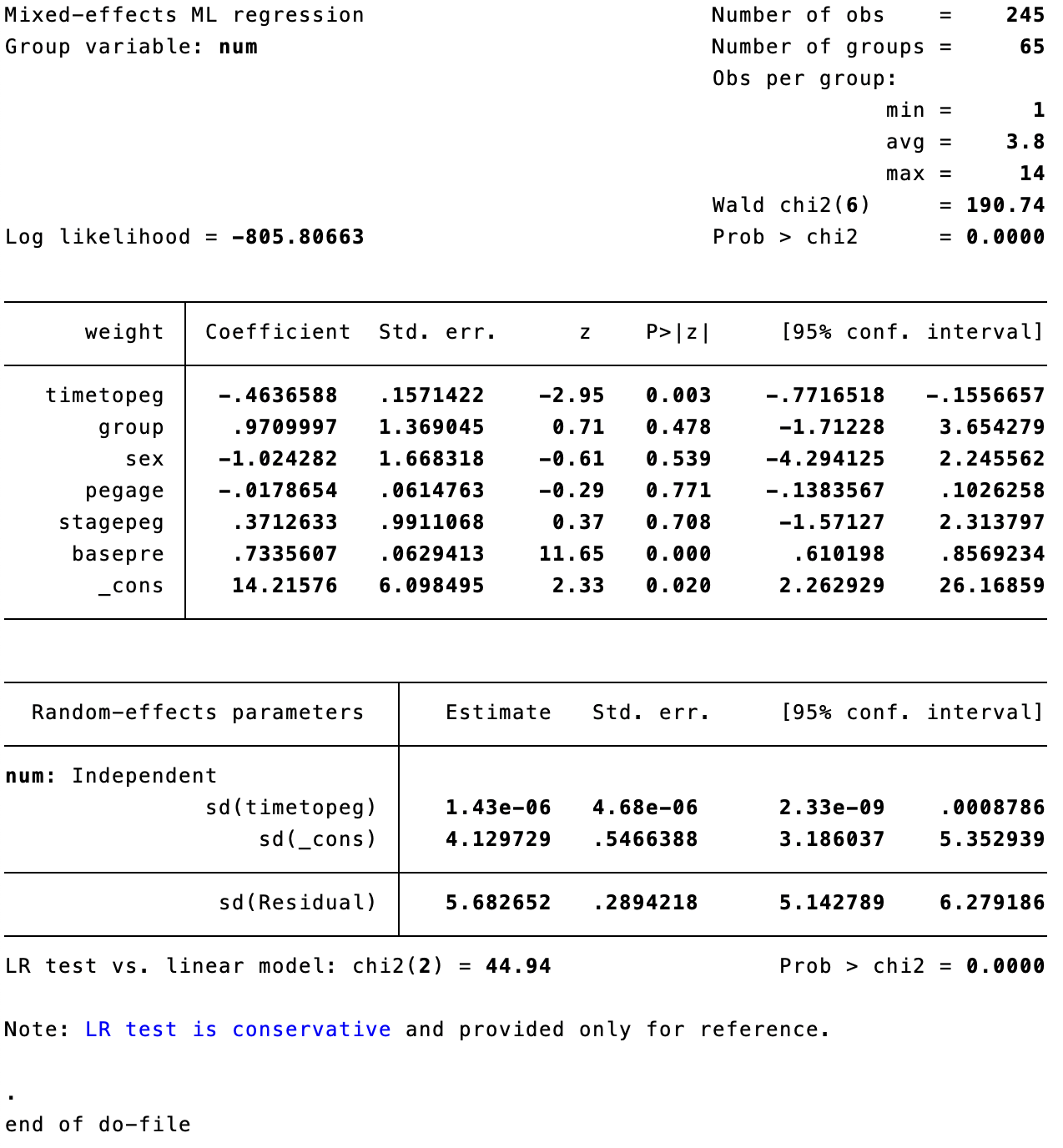


Legend: Weight (kg); timetopeg = time (years) before PEG insertion; group = PEG vs non-PEG; sex = male vs female; pegage = age (years) at PEG insertion/non-insertion; stagepeg = stage (Shoulson and Fahn staging) at PEG insertion/non-insertion; basepre = baseline weight (kg, first weight recorded).

**Supplemental Table 2:**

**Mixed effects regression model output for after PEG insertion**


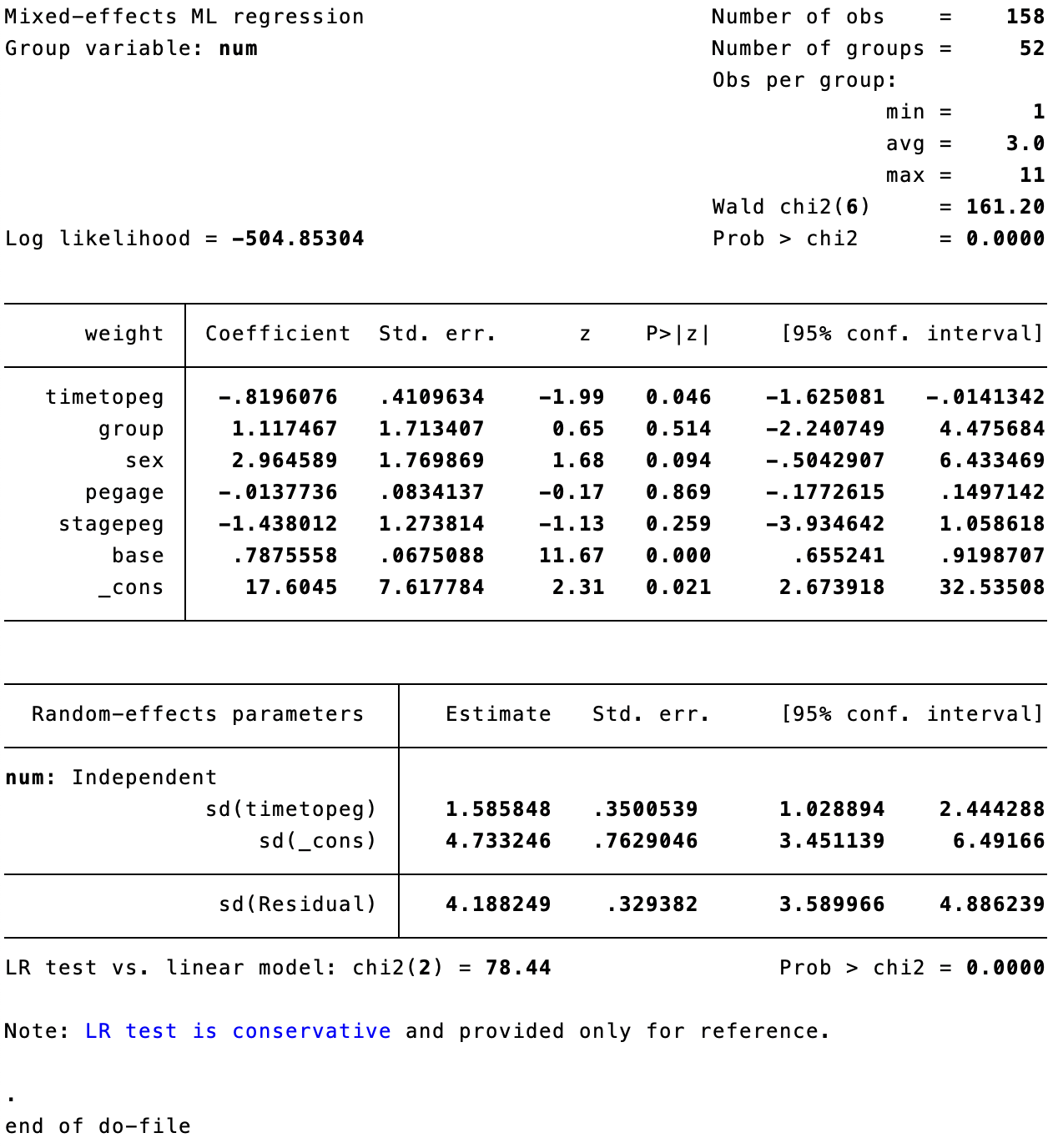


Legend: Weight (kg); timetopeg = time (years) after PEG insertion; group = PEG vs non-PEG; sex = male vs female; pegage = age (years) at PEG insertion/non-insertion; stagepeg = stage (Shoulson and Fahn staging) at PEG insertion/non-insertion; basepre = baseline weight (kg, weight at PEG insertion where available, otherwise imputed – see Methods section for details).
